# Review: Biomaterial systems to resolve brain inflammation after traumatic injury

**DOI:** 10.1063/1.5023709

**Published:** 2018-05-24

**Authors:** Francesca L. Maclean, Malcolm K. Horne, Richard J. Williams, David R. Nisbet

**Affiliations:** 1 Laboratory of Advanced Biomaterials, Research School of Engineering, The Australian National University, Canberra, ACT 2601, Australia; 2 Florey Institute of Neuroscience and Mental Health, University of Melbourne, Parkville, Australia; 3 Department of Medicine, University of Melbourne, St. Vincent's Hospital, Fitzroy, Australia; 4 School of Engineering, RMIT University, Melbourne, VIC 3001, Australia; 5 Biofab3D, Aikenhead Center for Medical Discovery, St. Vincent's Hospital, Melbourne, VIC 3065, Australia

## Abstract

The inflammatory response within the central nervous system (CNS) is a tightly regulated cascade of events which is a balance of both cytotoxic and cytotrophic effects which determine the outcome of an injury. The two effects are inextricably linked, particularly in traumatic brain injury or stroke, where permanent dysfunction is often observed. Chronic brain inflammation is a key barrier to regeneration. This is considered a toxic, growth inhibitory mechanism; yet, the inflammatory response must also be considered as a mechanism that can be exploited as protective and reparative. Repurposing this complex response is the challenge for tissue engineers: to design treatments to repair and regenerate damaged tissue after brain insult. Astrocytes are important cells within the CNS which play a key role after traumatic brain injury. A comprehensive understanding of their functions—both cytotrophic and cytotoxic—will enable designed materials and drug delivery approaches for improved treatment options post traumatic injury. Understanding, evaluating, and designing biomaterials that match the healthy neural environment to temporally alter the inflammatory cascade represent a promise neural tissue engineering strategy to optimise repair and regeneration after injury.

## INTRODUCTION

Repairing neural tissue after damage from injury or disease is a complex and yet unmet challenge. The spontaneous regeneration of neurons or axons in the adult brain is limited because of not only insufficient neurogenic potential but also nascent cells that can lack the environmental cues and signals that guide survival, differentiation, and reconnection.^1^ Many of these cues are provided by the extracellular matrix (ECM), native cells (astrocytes and microglia), and a range of both pro- and anti-inflammatory cytokines. The inflammatory response of the brain to injury is also complicated, and while it does have a reparative phase, this does not transition to full functional repair and regeneration^2^ such as that occurs in many other organs in the body, such as the skin^3^ or liver.^4,5^ When the blood-brain-barrier (BBB) is ruptured in penetrating brain trauma, there is an ingress of blood, fibroblasts, and other participants in inflammation (including macrophages, circulating white blood cells, and systemic cytokines). This complicates the inflammatory response in the brain over one that follows injury or cell death within an intact BBB. This presents a challenge for tissue engineers because engineering constructs designed to assist repair must be delivered via a penetrating brain injury—creating a traumatic injury in the delivery of the treatment itself. Nevertheless, there is a temporal profile in all injury responses where the initial phase is to attenuate haemorrhage and then remove foreign matter and necrotic debris. There may be advantage in attenuating this inflammatory cascade and subsequently enhancing the latter reparative stage, which in the brain is present but somewhat muted. Therefore, the damage (and response) to the implantation process must be considered when assessing the value and contribution of the constructs under consideration.

Within this context, we review the application of newly developed biomaterials for traumatic brain injury (TBI), with a particular focus on the need for temporal attenuation of the inflammatory response after penetrating brain injury to exploit this transition from defending the brain to repairing it. Initially, to contextualise the biological requirements, the inflammatory cascade and its associated events are introduced. Then, the complexity of central nervous system (CNS) inflammation, the associated impact on design criteria for biomaterials to assist in its resolution, and strategies that are used to improve outcomes of treatment strategies are discussed, and finally, perspectives on the future direction of the field are given.

## THE INFLAMMATORY RESPONSE

Inflammation is a constantly active, tightly regulated mechanism of a healthy body, which is also recruited following injury or with immune triggers, including infection. Following injury, the initial function of inflammation is to minimise tissue damage and maintain the integrity of the undamaged surrounding tissue, through bleeding, heat, immune cell invasion, scar formation, and eventually remodelling.

The degree and nature of the inflammatory response are related to the structures which are damaged, vascular damage, the degree of necrosis, and/or the presence of foreign materials. Vascular damage elicits a haemostatic response primarily directed at minimising blood loss;^6^ yet, it may also compromise cell viability and lead to further swelling. The entry of phagocytic cells is triggered by cytokines released due to the initial loss of tissue integrity, which generally occurs early in the inflammatory response. Phagocytic cells clear necrotic materials from primary or secondary injury as well as extraneous materials that may enter with the penetrating wound.^7^

These inflammatory actions, whilst crucial to tissue repair, can spread to the adjacent healthy tissue and cause secondary cell damage and death unless contained. The early response includes recruitment of fibroblasts to surround the region and prevent the egress of cytokines, blood, and other cytotoxic events into the surrounding healthy tissue. This fibroblast response leads to the deposition of ECM molecules which provide a scaffold for the process of repair of tissue integrity. This organised ECM later becomes scar tissue which in the absence of recurrent inflammation will resorb and be replaced by normal tissue. In the brain, the transition from the initial protective inflammatory phases to the reparative phases where new tissue is constructed and remodelled is not as successful. The reasons behind this insufficient transition of phases are at present not well understood, yet present a valuable therapeutic target.

Key to this puzzle is the response of a native cell type, particularly astrocytes post injury, which undergo proliferation limited to the lesion penumbra,^8,9^ experiencing significant hypertrophy, and increase their expression of intermediate filament proteins including glial fibrillary acid protein (GFAP), nestin, and vimentin in response to injury.^10^ This phenotype of astrocytes is referred to as “reactive,” as compared to the physiologically active astrocyte phenotype in healthy tissue. These reactive astrocytes can entangle their filamentous processes to form a physical barrier (termed either the astrocyte or glial scar) around the lesion core that not only inhibits immediate axon regeneration after injury but also restricts the migration of inflammatory cells into viable tissue preventing secondary degeneration.^11^ In recent years, there has been a shift in the perception that astrocytes are either good (“quiescent”) or bad (“reactive”), with the recognition that astrocytes, whilst they may be reactive after injury, serve essential functions including maintenance of the BBB, supporting neuronal survival, and preventing excessive inflammatory cell infiltration. We direct the interested reader to consider the following for a view of astrocytes beyond this binary sense.^2,8,12^

The interconnected mechanisms of cells involved in the CNS inflammatory response increase the complexity of developing tissue engineering approaches for repair and thus are currently a problematic aspect crucial to regeneration. Although microglia, macrophages, and other infiltrating inflammatory cells all play a role after injury, they are the prevalent barrier of the astrocytic scar which is the major hurdle to overcome. Therefore, strategies must be developed to reduce or remove this scar.

## THIRD-GENERATION BIOMATERIAL STRATEGIES TO CONTROL THE INFLAMMATORY RESPONSE AFTER TRAUMATIC BRAIN INJURY

Biomaterials have long been of interest in medical treatments. Initially, the focus was on providing mechanical and structural support in age-related ailments such as hip and dental replacements; yet, these materials were pragmatic solutions, not designed to replace more complex living tissues.^13^ Advances in the understanding of the biology informed second-generation biomaterials to exert control on the surrounding environment.^14,15^ Third-generation biomaterials are designed to (i) provide instructive cues at the molecular level, (ii) respond to the physiological environment, and (iii) degrade over time, with the goal of leaving nothing but newly regenerated host tissue. As such, this class of biomaterials has generated optimism for TBI/neural tissue engineering applications to influence and instruct host tissue to achieve repair and regeneration including targeted manipulation of the inflammatory response. Here, we discuss the overarching strategy for achieving functional recovery after injury and then identify those approaches that modify the inflammatory response.

### Design criteria for brain repair and controlling inflammation

A series of design criteria can therefore be established for an “ideal” biomaterial for the treatment of traumatic brain injury. The material should

1. Be easily deliverable to the site of injury and once implanted provide the physical support for the surrounding tissue.
2. Match the biochemical environment of the brain (water content, pH, etc.).
3. Match the biophysical environment of the brain (elastic modulus, porosity, etc.).
4. Encourage cell infiltration into, through, and across the lesion site.
5. Match the three-dimensional (3D) architecture of the brain's extra-cellular matrix on a biologically relevant length scale.
6. Selectively deliver biological cues to the surrounding tissue at time points based on the different stages of the inflammatory response.

We have established these criteria for their importance in attenuating the inflammatory response after injury. Physical support (criteria 1) is crucial as further tissue collapse after the initial swelling post-injury can exacerbate the existing inflammatory response. Matching the modulus of the brain (criteria 3) will also avoid the development of a foreign body reaction (FBR) from microtrauma that arises from modulus mismatch, such as in the case of implanted electrodes.^16,17^ Finally, the time controlled delivery of biological cues (criteria 6) is of great importance in an inflammatory context. It is important to allow the inflammatory astrocytic response to exist for a short term; however, the persistence of this reaction needs to be attenuated, which could be achieved through temporally controlled biological intervention, after which cell infiltration can be supported (criteria 4). This is an important criterion, and while many studies have reported limited nutrient exchange and ingrowth into hydrogels, we have recently advanced the field with the implantation of human cortical progenitor cell grafts within our novel self-assembling peptides (SAPs) in a stroke brain.^18^ We demonstrated progressive motor function improvement in the rat brain over 9 months when compared to the cell only or scaffold only control. The animals that received the combination of cells and SAP had significantly less cortical atrophy, with larger grafts, greater neuronal differentiation, and significantly enhanced electrophysiological properties demonstrating that the neurones were fully integrated. Importantly, we observed significantly angiogenesis within the grafts that were green fluorescent protein (GFP), indicating that the blood vessels were host derived and highlighting that endogenous cells readily infiltrated the hydrogel. Additionally, we observed significant graft derived (GFP+) innervation in the ipsilateral and contralateral hemispheres, highlighting the ability of axons to grow out of the hydrogel and integrate within the brain. While this work is particularly promising, we concede that it is yet to be an idealised material, which will require significant, further collaborations occurring between chemists, engineers, and biologists. Here, we present a review of efforts to develop such biomaterials.

### Biomaterials to provide physical support to the lesion site

A penetrative TBI (from say a gun-shot or stab) results in an odd-shaped lesion site in the brain that the natural healing mechanisms are unprepared to deal with. This primary injury causes severe tissue damage, followed by swelling which can necessitate a craniotomy to reduce the swelling. Once the swelling subsides, structural support to the damaged area is necessary to avoid tissue collapse. Therefore, any treatment strategy for TBI must offer physical support to the lesion site. If a biomaterial can adequately offer physical support and be implanted then it is a potential candidate for physical support; examples include electrospun nanofibrous scaffolds^19^ and a variety of hydrogels. In order to minimise the damage caused by implantation, materials that can flow and be injected (particularly if they are shear-thinning),^20^ are easily administered without being overly destructive. It should be noted that due to TBI, the blood brain barrier will most likely be disrupted, and therefore, maintaining its integrity is of secondary concern.

**FIG. 1.**
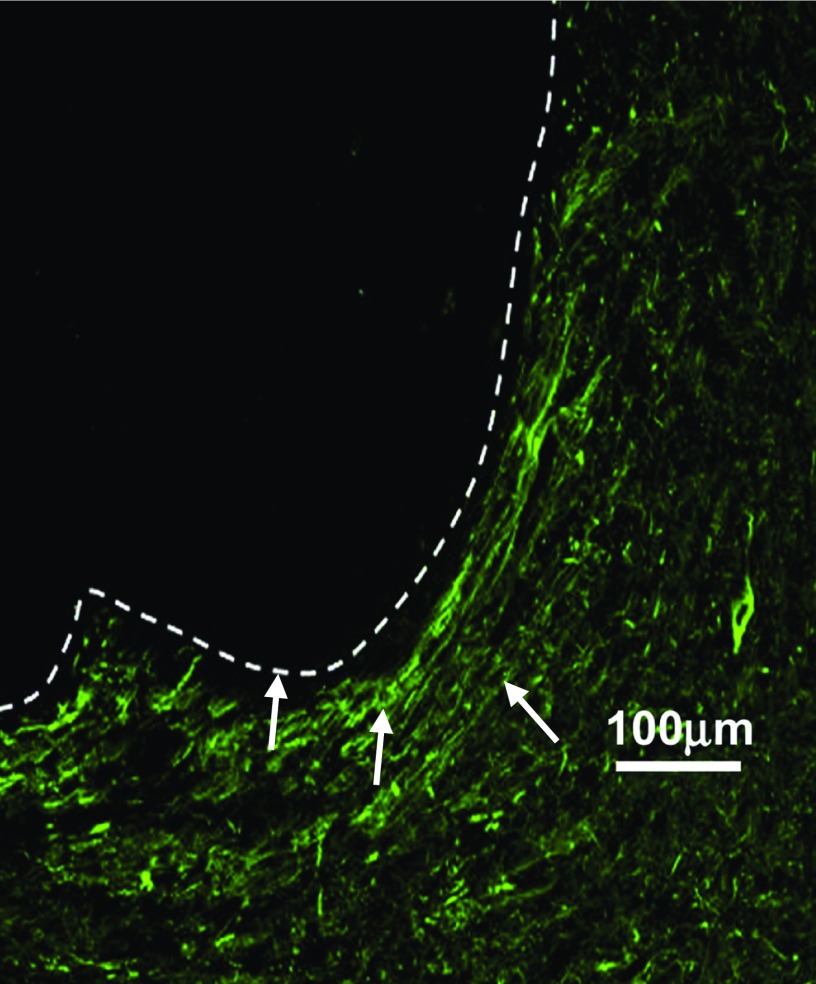
White arrows show neurites growing around an electrospun scaffold implanted in the brain. This demonstrates an inability of electrospun scaffolds to form an intimate contact with the parenchyma and endogenous neurites within a lesion cavity. Adapted with permission from Nisbet *et al.*, Biomaterials 30(27), 4573 (2009). Copyright 2009 Elsevier.

Electrospun nanofibre scaffolds are interesting biomaterial candidates as they can be easily fabricated from biocompatible and biodegradable materials, also mimic the nanofibrous architecture of the ECM, possess a high surface area-to-volume ratio, and can be functionalized to provide instructive cues to cells through the immobilization of growth factors and other biologically relevant cues.^21–25^ The 3D architecture of nanofibres has been previously shown to shift the biological profile of astrocytes to that of more cytotrophic nature *in vitro*,^26^ which could have potential value *in vivo,* altering astrocytic phenotype after injury. Randomly aligned poly(ε-caprolactone) (PCL) scaffolds significantly reduced microglia and astrocyte numbers after implantation into the caudate putamen of male Wistar rats compared to the wire control.^19^ This was important as it highlighted that there was no significant inflammation, from either astrocytes or microglia, that arose from the acidic degradation production of the electrospun PCL, with the inflammatory cascade being statistically the same as the platinum needle sham at all time points tested. After 60 days, this scaffold also supported the infiltration of neurites, compared to neurite growth which was directed around the scaffold when nanofibres were partially aligned. Nanofibre scaffolds have also been investigated for their ability to deliver biological cues *in vivo*. For example, when primary cortical neural cells were transplanted adjacent to PCL scaffolds with glial-cell derived neurotrophic factor (GDNF) immobilized to the surface, their survival, proliferation, and neurite outgrowth were enhanced.^27^ These studies demonstrate the utility of nanofibre scaffolds in attenuating the inflammatory response of the brain, as well as facilitating cell growth. However, a shortcoming of electrospun nanofibres, as previously highlighted,^12,28^ is the geometrical constraint associated with possessing a sheet-like structure on the macroscale. These sheets can be rolled upon themselves to form an injectable cylinder; however, this structure cannot form an intimate contact with surrounding tissue in an odd-shaped lesion, as is commonly found in TBI (Fig. 1) although they would be of interest for grafting over damaged nerves or in spinal cord injuries.^29^ Therefore, to satisfy the criteria of providing physical support to the lesion, injectable hydrogels are ideal candidates, as they can flow to fill the lesion site and prevent further tissue collapse after injury.

Nanofibre scaffolds are of interest, as they have significant promise for the systemic *in vitro* investigation of astrocytes and their inflammatory behaviour. Given their capacity to provide a 3D nanofibrous environment, present biological cues,^19,22,29^ and alter astrocyte phenotype and behaviour,^26,30,31^ eletrospun nanofibrous scaffolds can be used to better understand astrocytes and their complex behaviour *in vitro*. Additionally, since *in vitro* use of hydrogels as 3D environments can present challenges (for instance, with handling, degradation, and imaging), nanofibre scaffolds can facilitate the shift of *in vitro* investigation from 2D to 3D environments.

### Matching the modulus of the brain

**FIG. 2.**
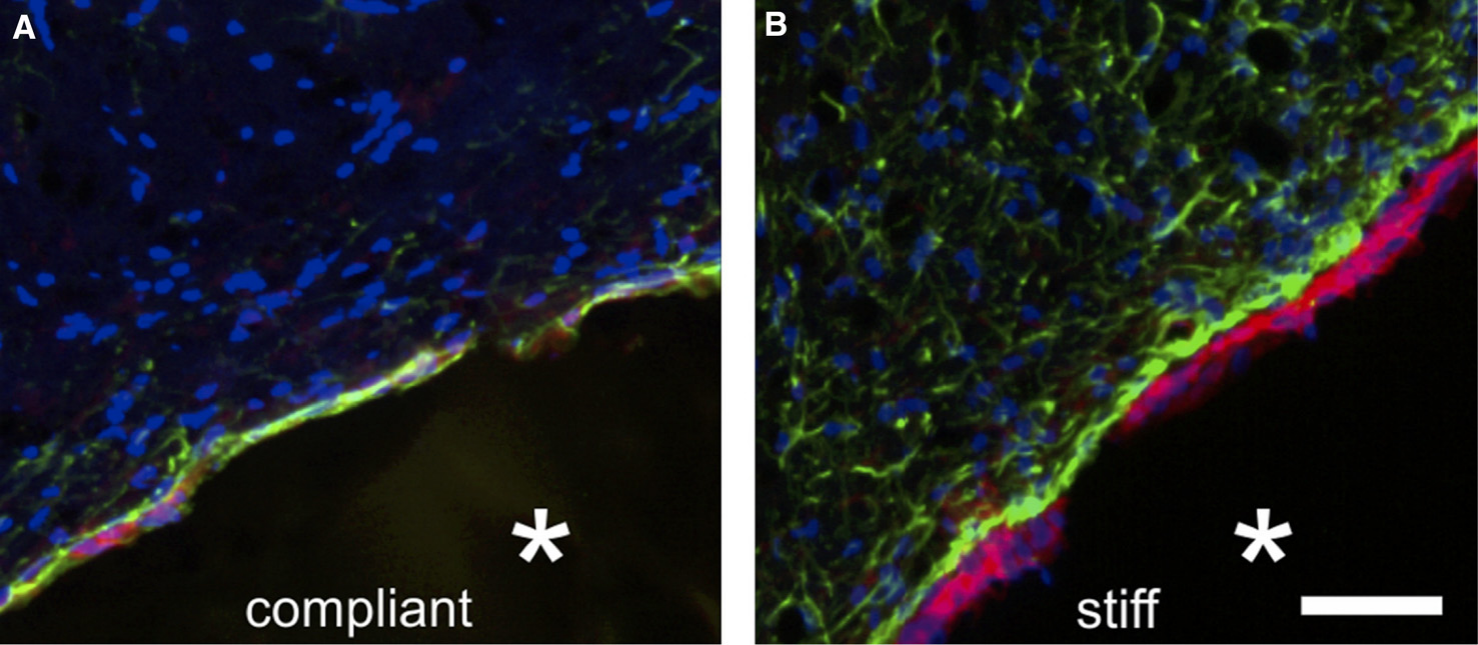
The immune response resulting from a compliant (0.1 kPa) and stiff (30 kPa) hydrogel 3 weeks post implantation. This figure highlights a comparative increase in the foreign body reaction for the 30 kPa hydrogel. Cells are marked with GFAP (green for astrocytes), OX42 (red for microglia), and Hoechst (blue for cell nuclei). Reproduced with permission from Moshayedi *et al.*, Biomaterials 35(13), 3919 (2014). Copyright 2014 Elsevier under a Creative Commons Attribution License.

From a material point of view, the brain can be considered an elastic solid. Measurements of the Young's modulus of the brain show heterogeneity, and depending on the region, it varies between ∼0.1 and 2 kPa and also varies with white and grey matter.^32,33^ It is vital that any biomaterial system selected for TBI treatment can approach the Young's modulus of targeted brain tissue to minimise microtraumas associated with elastic mismatch, and given the varying nature of the brain's elastic properties, a system which can be mechanically tuned is also ideal. Modulus mismatch can be the downfall of many medical devices, due to the mechanosensitivity of microglia and astrocytes. Not only does the implantation procedure cause an inflammatory response (as it is essentially another form of penetrating injury), but also modulus mismatch initiates a FBR (for example, as a result of constant microshearing), and so, microglia and astrocytes become reactive and seal off the implant. This is an important consideration, as the formation of the astrocyte scar as part of the FBR prevents the implant from having contact with host tissue to enable regeneration. This was clearly demonstrated when polyacrylamide (PAA) gels, at either 0.1 kPa (compliant) or 30 kPa (stiff), were implanted into Sprague-Dawley rat brains, Fig. 2.^34^ There was an increased astrocyte and microglia response when the stiff (30 kPa) hydrogel is implanted, highlighting the importance of the consideration of the modulus of biomaterials when designing a treatment strategy for TBI.

Given their ability to fill a void (compared to nanofibre scaffolds), hydrogels will be primarily considered when assessing biomaterials to match the modulus of brain tissue, as they can also satisfy the previous criteria of providing physical support.^35,36^ Many hydrogel systems not only match the modulus of the brain tissue but also have tunable mechanical properties, making them ideal tissue engineering constructs for TBI. Mechanical properties can be tuned through various routes, including cross-linker or precursor concentration and fibre density. Previously, mechanical properties have been of great interest for researchers in controlling the differentiation, proliferation, and morphology of stem cells,^33,37^ which has led to a large number of hydrogels being investigated for their tunable mechanical properties.

A commonly used hydrogel for tissue engineering applications extracted from brown algae, alginate, has a modulus which is dependent on the molecular weight and cross-linking. Alginate consists of linear co-polymers comprising blocks of α-l-guluronate (G) and (1,4)-linked β-d-mannuronate (M) residues, with the G-blocks being key determinants of mechanical properties through the co-polymer length and molecular weight.^38^ Ionic cross-linking of alginate is a common gelation method, with the divalent calcium ion being a popular ion choice. Calcium ion cross-linking can be controlled through the use of low-solubility solutions which result in a slower release of Ca^2+^ and thus a more controlled gelation and consequently more uniform mechanical properties. To match the modulus of brain tissue, the alginate and Ca^2+^ concentration can be optimized within the gelation process.^39^ Ionic cross-linking limits the stability of the hydrogel over longer time periods, which could be advantageous for brain repair if the release of the divalent ions (Ca^2+^), and thus hydrogel dissolution, could be controlled. Alternatively, covalent cross-linking can offer a greater degree of control over the mechanical properties of alginate; however, these cross-linking reactions can have unreacted and cytotoxic reagents, and so, further work is required to remove these cytotoxic agents from the covalently cross-linked alginate gels.

Hyaluronic acid, a polysaccharide abundant in the native ECM, has been extensively investigated for its tunable mechanical properties that are dependent on the molecular weight, chemical crosslinking, and ultraviolet (UV) cross-linking. These determining variables can also be combined to optimize hydrogel formation and modulus. For example, chemical cross-linking of methacrylated hyaluronic acid with a dithiol cross-linker (via a Michael addition mechanism) coupled with UV cross-linking resulted in a consistent peak modulus of the hydrogel (∼100 kPa). However, without UV cross-linking, hydrogel modulus increased over two orders of magnitude with the increasing cross-linker concentration, reaching ∼100 kPa with 100% crosslinker consumption.^40^ Modification of hyaluronic acid with photo-crosslinked methacrylate groups can yield a range of moduli, matching that of a neonatal brain to that of an adult spinal cord, demonstrating the utility of this system's tunable modulus.^41^

A synthetic hydrogel with significant potential for enhanced functionality is poly(ethylene glycol) (PEG). Various ways to crosslink PEG hydrogels have been extensively reviewed elsewhere.^42^ Increased cross-linking of star-PEG/heparin hydrogels increased the associated storage modulus, accompanied by a reduction in hydrogel hydration, demonstrating the impact of modulus on water uptake (a key characteristic of hydrogels for tissue engineering).^43^ Additionally, modification of PEG hydrogels with biological moieties such as the cell adhesion sequence, arginine-glycine-aspartic acid (RGD), can also affect the modulus. PEG hydrogels cross-linked with varying concentrations of PEG-dithiol (PEG-SH) showed an increase in modulus when the sequence RGD was attached to the hydrogel.^44^ These relationships between modulus and other material properties demonstrate the need for the optimisation of cross-links, water uptake, biological functionalization, and modulus when designing a tissue engineering construct for brain repair.

All these hydrogels present desirable mechanical properties for use in treating traumatic brain injury; however, they are unable to mimic the nanofibrous structure of the ECM (criteria 6), and as such, the remainder of this review will focus on the use of nanofibrous hydrogels or composite materials which can satisfy the same criteria. Such nanofibrous hydrogels are a more recently developed class of hydrogels, self-assembled peptide (SAP) hydrogels. They form fibrils within a hydrogel and can provide a variety of biological functionalities through either the peptide sequence or additional functionalization. The modulus of these SAPs is dependent on the peptide sequence and concentration, as well as the gelation mechanism. The formation of the nanofibres determines the bulk physical properties of the hydrogels: for example, peptide amphiphiles (PAs) such as C_16_A_4_G_3_S(P)KGE-COOH (termed PA-1) can form a hydrogel at concentrations as dilute as 0.5 wt. %, forming nanofibres 6–7 nm in diameter.^45^ PAs consist of a short hydrophobic alkyl chain, attached to a short hydrophilic (relative to the alkyl chain) peptide sequence, which self-assemble to form a hydrogel with high-aspect-ratio nanofibers.^46^ This self-assembly is driven by the thermodynamic incompatibility between the different regions of the chain and triggered by charge neutralization via the addition of metal ions.^47^ These PAs can aggregate to form different morphologies including spherical micelles, cylinders, and even bilayer vesicles.^48^ Gelation and initial solution pH have been shown to be influencers of PA modulus. The modulus of PA-1 reaches a plateau when gelled with Ca^2+^ concentrations of 20–30 mM, due to a saturation of metal ion-PA interactions, whilst the initial pH of the solution influences the gel formation, reaching a maximum modulus of ∼1000 Pa above pH 9. This is a result of deprotonation, which creates further negative charges that interact with Ca^2+^, stabilizing interfibre bonds and increasing the density of fibre crosslinks and thus modulus.^45^ RADA16-1 is another synthetic amphiphilic peptide; however, it has a comparably low modulus, which has been noted as a limitation of its use.^49,50^ Modification with the addition of a peptide motif inspired by spider silk, GPGGY, tripled the modulus compared to unmodified RADA16-1; however it still remained low (∼20 Pa). RADA16-1 is also limited by its low pH, which resulted in increased inflammation when transplanted into a spinal cord injury without at least 7 days “pre-treatment” where the pH was neutralized using culture medium.^51^ These issues highlight the need for further development of the RADA16-1 system before it is as competitive as other SAP systems for treating traumatic brain injury.

A similar modulus dependence on the gelation mechanism as seen in PA-1 is also observed in the Fmoc-capped self-assembled peptide hydrogels. Short bioactive peptide sequences are attached to the aromatic Fmoc-group, which undergoes self-assembly in response to a pH-switch. The morphology and modulus of Fmoc-FRGDF can be manipulated via the final ionic strength and the rate of pH change during gelation, with an increased ionic strength associated with faster gel formations and thus increased stiffness of the hydrogel.^52^ Additionally, using glucono-δ-lactone to replace hydrochloric acid in the pH switch decreased the gelation rate, resulting in a decreased modulus compared to hydrogels formed using hydrochloric acid. The addition of D-residues in Fmoc-IKVAV, Fmoc-DIKVAV, Fmoc-DDIKVAV, and Fmoc-DDDIKVAV was associated with increasing stiffness,^53^ demonstrating that the peptide sequence, ionic strength, peptide concentration, and the rate of gel formation can be used to tune the moduli of Fmoc-SAPs to match that of brain tissue.

Matching and ideally tuning the moduli of these nanofibrous hydrogels is vital in the development of a biomaterial system to treat traumatic injury, and here, we have presented many nanofibrous hydrogels which can do so. Therefore, these materials should be the focus of future research effort to further develop their functionality and eventual deployment as a TBI treatment.

### Controlled degradation of biomaterial candidates

The mechanical properties of material candidates are a crucial design consideration to avoid a FBR, and since temporal control of the surrounding environment is required, a material that can change in a timely manner to facilitate cell infiltration or deliver a repertoire of signals is also ideal. The relationship between moduli and hydrogel degradation is an important consideration for these material systems to achieve functional repair and regeneration at the lesion site.

There are a variety of mechanisms that can be used to control the degradation of a biomaterial. Some are inherent to the material itself: for example, PCL and poly(l-lactic acid) nanofibres are degradable via the hydrolysis of the ester linkages, whilst other mechanisms can be included within the material to impart controllable degradation. Other mechanisms are an addition to the biomaterial system to overcome a natural degradation limitation. For example, although alginate has been extensively investigated for both *in vitro* and *in vivo* use, it is not enzymatically degraded by mammals. With previous research demonstrating enhanced vascularization and tissue integration of cells transplanted within readily degradable materials, mechanisms to encourage this degradation have been investigated.^54,55^ Encapsulation of alginate-lyase within poly(lactic-co-glycolic acid) (PLGA) microspheres (which are then embedded within the alginate hydrogel) enabled a tunable enzymatic degradation of the alginate hydrogels and facilitated a greater rate of proliferation when neural progenitor cells (NPCs) were also encapsulated.^56^ This controlled degradation mechanism via microspheres could be introduced within the fibrous hydrogel systems to aid in degradation.

Proteolytic degradation can also be employed to achieve cell-mediated degradation. For example, a peptide sequence can be included in self-assembled peptides or PEG crosslinks that are sensitive to matrix metalloproteinases (MMPs).^57–60^ However, such control over material degradation may be limited in some cases. Where degradation cannot easily be controlled, without compromising material properties, it may be of interest to develop a composite material. This composite system has been preliminarily investigated using RADA16 functionalised with RGD which was chemically crosslinked to PEG to form a hydrogel with modulated mechanical properties and a nanofibrous structure, demonstrating the achievable nature of such a composite.^61^ These degradation mechanisms could be optimized to achieve temporal delivery of cues to modify the inflammatory response and facilitate cell infiltration.

### Modifying the inflammatory response via spatiotemporal delivery of cues

As previously established the inflammatory astrocytic response after injury is complex and necessary for repair. The cytotoxic effects of this response are here considered to be the persistent nature of the astrocyte scar around the lesion site. Thus, when designing a biomaterial construct to resolve this inflammation, temporal control of this response is required. After a stab injury, astrocyte numbers have been found to peak at 7 days (although their particular phenotype/sub-phenotype of reactivity is unknown).^62^ Within the first two weeks post-injury, astrocytes proliferate and form the scar, with scar remodelling occurring from 3 weeks onwards, where it can remain persistent in severe injuries.^2^ Therefore, 3–4 weeks post injury is the ideal time-point to begin the delivery of therapeutic agents to resolve the inflammatory process and provide a growth-supportive environment.

**FIG. 3.**
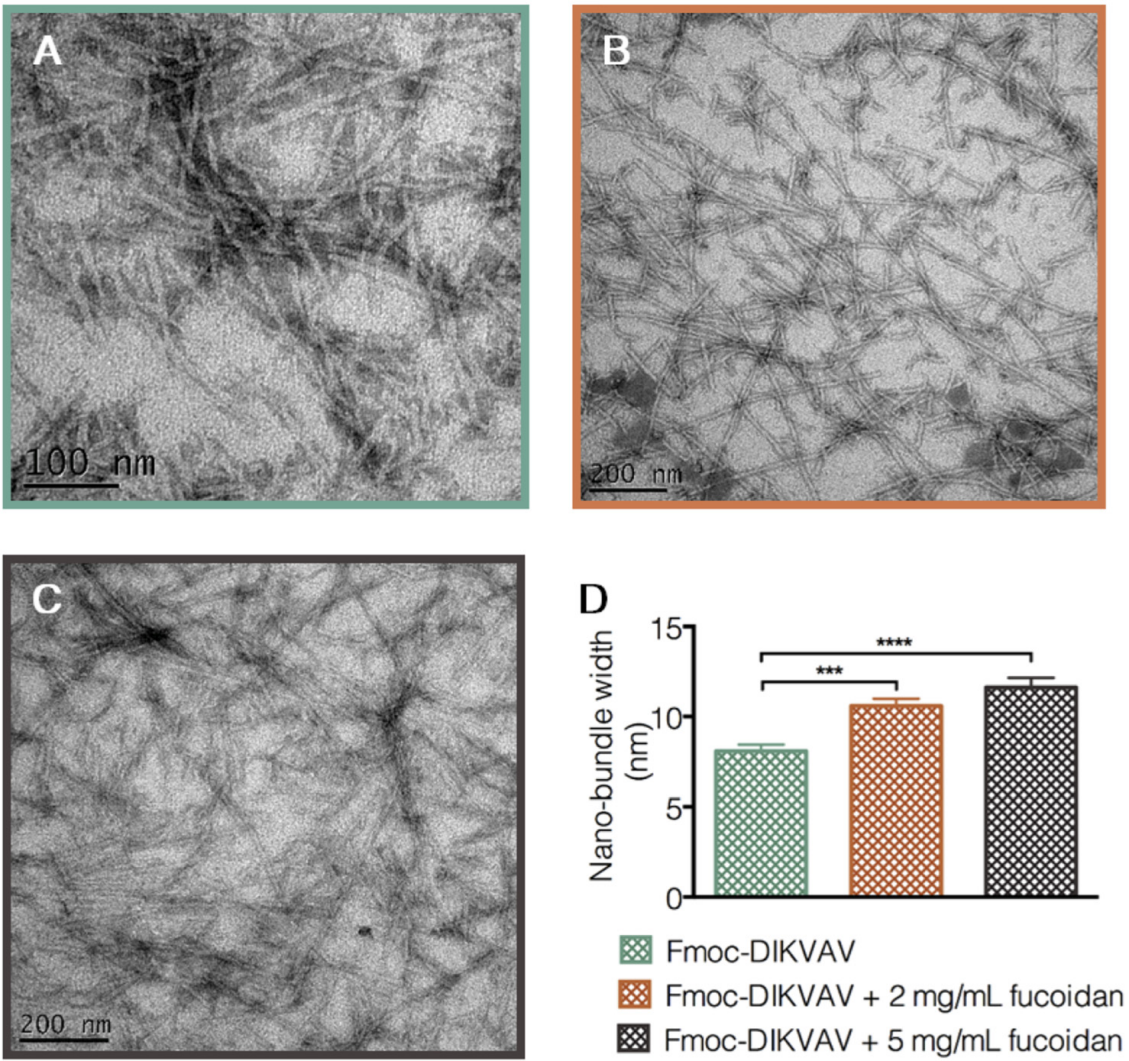
Transmission electron micrographs that confirm the π-β assembly of a novel Fmoc-DIKVAV hydrogel. This hydrogel presents the bioactive sequence derived from laminin (IKVAV) at high density of the fibril surface. (a) presents the Fmoc-DIKVAV hydrogel, (b) Fmoc-DIKVAV + 2 mg/ml fucoidan, and (c) Fmoc-DIKVAV + 5 mg/ml fucoidan, and (d) shows the tendency for fiber bundling with the increasing fucoidan concentration. Errors are reported as *** p < 0.001 and ****p < 0.0001. Reproduced with permission from Maclean *et al.*, ACS Biomater. Sci. Eng. 3(10), 2542 (2017). Copyright 2017 American Chemical Society.

**FIG. 4.**
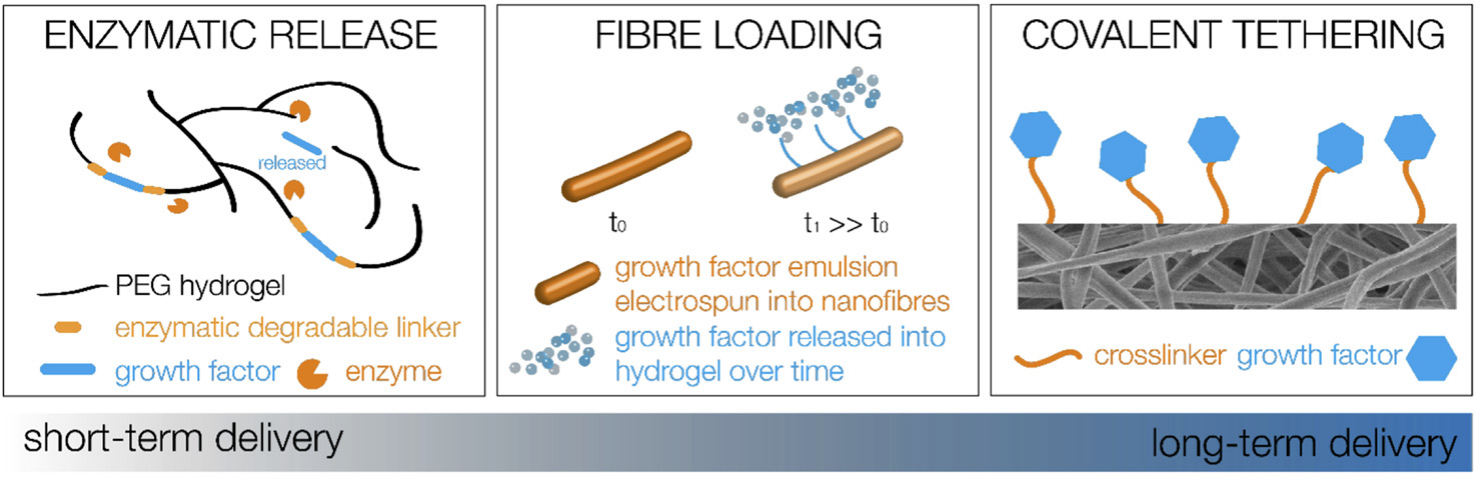
Varying potential material solutions for time resolved delivery of therapeutic agents within and from the implanted biomaterials.

Mechanisms which can be employed to achieve temporal delivery of therapeutic agents, other than material degradation as discussed above, include diffusion and enzymatic-triggered delivery. One avenue for delivery is the incorporation of molecules via co-assembly or mixing within the hydrogel construct, which has been achieved with the anti-inflammatory fucoidan without degrading the structure as shown in Fig. 3 (Refs. 62 and 63) as well as brain-derived neurotrophic factor (BDNF) and GDNF^64^ within various Fmoc-SAP systems. A delayed release of BDNF was achieved via modification with chitosan, demonstrating that either the delivery mechanism or the therapeutic agent itself can be modified to alter the delivery time. Incorporating drug delivery vehicles within a hydrogel construct would provide two diffusive barriers to release—first, out of the delivery vehicle and then from within the hydrogel to the surrounding tissue. Examples of such delivery vehicles include microspheres and nanoparticles: nanoparticles containing flavopiridol have reduced cavitation and pro-inflammatory cytokine expression after spinal cord injury.^65^ However, it was found that these nanoparticles only had sustained release of 3 days *in vitro*, and so, including them within a hydrogel would delay the release of such a therapeutic agent to a more desirable time-point. A similar delivery mechanism can be achieved using electrospun nanofibres, where the therapeutic agent is included within the electrospinning emulsion, and the subsequent nanofibres are cut into short nanofibres and included within a hydrogel. The diffusion from these fibres is visualized in Fig. 4, and when combined within a hydrogel, they mitigate a burst release of drug and delay the subsequent release.^66^

Another mechanism which can be combined to provide temporal release and degradation is enzymatic-triggered release of therapeutic agents from the hydrogel itself. This has been elegantly demonstrated with a proangiogenic peptide included within the cross-links of PEG, which was released upon exposure to host matrix metalloproteinases (MMPs).^60^ This resulted in the bulk degradation of the material and significant angiogenesis at the site of hydrogel implantation, demonstrating the therapeutic utility of this system. To modulate the inflammatory response, this enzymatically responsive release could be used to deliver anti-inflammatory therapeutic agents if the enzyme-responsive linker could be tailored to enzymes that would be present at 3–4 weeks post injury.

Long term delivery of growth factors can be achieved through covalent immobilization on scaffolds, such as nanofibers,^22,29,67^ which can then be incorporated into a hydrogel as short fibres, to satisfy the previous criteria discussed. This would provide sustained and long term delivery of biological cues which would be of use when encouraging regeneration after scar resolution, however, in itself does not provide the ideal level of temporal control. Therefore, these short nanofibres would be valuable when incorporated as one of many components in a multifaceted system.

There are a variety of delivery mechanisms which can impart temporal control over therapeutic agent release; however, considerable investigation into what additional cues are the most effective in resolving the persisting scar needs to first be completed. What would be of interest are therapeutic agents that can impact the astrocyte morphology, which is linked with the astrocyte phenotype. The family of Rho-GTPases regulate the assembly of cytoskeletal processes which determine the cell shape and contractility, which could be valuable for changing astrocyte morphology/phenotype, and the mechanotransductive signals RhoA and CdC42 are activated when neural stem cells (NSCs) respond to ECM stiffness (which we have previously discussed as a key consideration for a TBI treatment).^68^ In particular, Rho Kinase has been highlighted as a valuable therapeutic target for reactive astrocytes [its suitability for mediating astrocyte reactivity (after stoke) is comprehensively reviewed in Ref. 69)] as its inhibition has resulted in reduced lysophosphatidic-induced stress fibres and focal adhesions,^70^ as well as decreased F-actin but increased G-actin in mature primary astrocytes grown on nanofibre scaffolds, indicating a more cytotrophic phenotype of astrocytes.^30^ Contrastingly, when delivered with fibroblast growth factor (FGF), the sulfated polysaccharide extracted from sea cucumber, Haishen (HS), modulated astrocyte morphological transformation, increased cell proliferation, and was thus proposed as an adjuvant to induce astrocyte reactivity. These two examples demonstrate that changes in the astrocyte morphology can induce both physiologically active and reactive astrocytes, and thus, therapeutic agents need to be selected carefully. Astrocyte activity has also been modulated by the presentation of poly(d-lysine) (PDL) grafted to the thermally gelling hydrogel xyloglucan. After 60 days, the percentage area of both astrocytes (GFAP^+^) and neurites (SMI32^+^) were highest when the greatest amount of PDL was grafted to the xyloglucan hydrogel.^62^ The increase in both astrocyte and neurite infiltration with the PDL presentation and possibly an alteration in the astrocyte phenotype from reactive to growth-supportive suggest that saccharides would be a valuable avenue of investigation for the attenuation of inflammation after injury. Thus, we propose that the inclusion of anti-inflammatory saccharides to alter the astrocyte phenotype (and morphology) is a valuable avenue of investigation for the development of systems to resolve the astrocyte scar after TBI. This includes the presentation of galactose moieties such as lactobionic acid, for example, or the anti-inflammatory sulfated polysaccharide fucoidan. Fucoidan has induced apoptosis and the decreased expression of pro-inflammatory cytokines in cancer cells when delivered via Fmoc-FRGDF,^71,72^ whilst the neutral polysaccharide PPQN has suppressed nitrous oxide (NO) production and pro-inflammatory cytokine secretions in lipopolysaccharide (LPS) stimulated macrophages.^73^ These results highlight the potential for the inclusion of anti-inflammatory saccharides, which can then be included via a temporal delivery mechanism within the biomaterial constructs we have discussed here to develop an effective TBI treatment.

## CONCLUSION AND FUTURE PERSPECTIVES

Here, we have discussed the complexity of inflammation within the central nervous system and have specifically focused on evaluating current biomaterial systems for developing a treatment for TBI which can also impact the inflammatory response. Given the complexity, as well as the incomplete knowledge and characterization of the astrocyte response, tissue engineering solutions to TBI face many challenges. As such, we have presented tissue engineering design criteria for a solution to be used in TBI and explored the criteria that are of particular importance in addressing the inflammatory response. It is essential that any biomaterial system can fill an odd-shaped lesion void, forming an intimate contact with the surrounding tissue to prevent further tissue collapse. Thus, hydrogel systems were explored over nanofibrous scaffolds, as they can easily fill a void. In particular, hydrogels with modulus matching that of brain tissue, and ideally with a tunable modulus, are of particular interest to facilitate neural growth and mitigate a foreign body response.

**FIG. 5.**
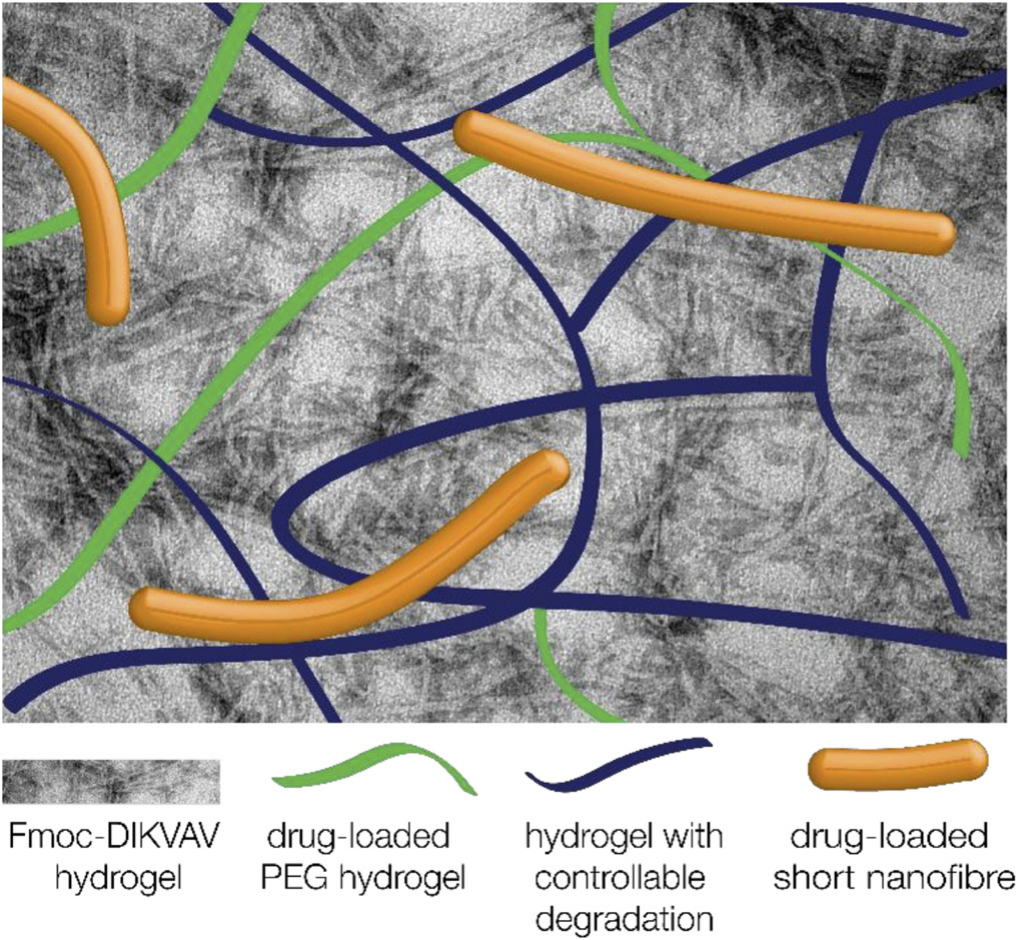
Composite biomaterial to provide mechanical, morphological, and biological cues with temporal control to improve functional repair after traumatic injury.

There are many systems possessing tunable moduli, dependent on the gelation mechanism and cross-linker, precursor, or fibre density. However, many of these systems do not satisfy key criteria for a TBI tissue engineering construct, which is to possess a nanofibrous architecture mimicking that of the brain. Thus, self-assembled peptides are of particular interest in developing a biomaterial treatment strategy for TBI. The modulus of these materials can be tuned, and their degradation can also be tuned via degradable peptide links or through the development of a composite hydrogel system with another material component with tunable degradation. The comprehensive characterization of material degradation and possibly the addition of controllable degradation mechanisms are necessary for the development of a successful, potentially resorbable biomaterial TBI treatment. Finally, we briefly discussed potential mechanisms to impart temporal delivery of therapeutic agents to resolve the persistent astrocyte scar to maximize their reparative functions after injury. Enzymatic-trigger or diffusive release as well as long-term immobilization of therapeutic factors is the potential delivery option; however, further research into the ideal factors is required. This would yield a biomaterial system with appropriate mechanical and morphological cues, as well as biologically relevant and temporal delivery of therapeutic agents to encourage growth supportive phenotypes in astrocytes, as shown in Fig. 5.

To enhance the success of tissue engineering constructs for TBI, we suggest that the investigation of composite material systems is the most promising avenue of investigation. Combining well-characterised hydrogel systems with tunable moduli and novel self-assembled peptide hydrogels as well as temporal delivery mechanisms will satisfy the tissue engineering criteria presented here for TBI treatment. By designing biomaterial systems that can satisfy the criteria for an *in vivo* treatment strategy, their *in vitro* investigation will be more valuable than current *in vitro* work conducted in 2D environments that fail to replicate ECM. During the development of a biomaterial system to treat TBI, the impact on astrocyte inflammation should be continually tested *in vitro* parallel to material development. We and others have previously discussed the importance of developing 3D cell culture environments in studying neuroinflammation,^12,74,75^ and similarly, we need to develop biomaterial systems that can be an effective treatment *in vivo*, which we can test first *in vitro*. It would be of value to combine these biomaterial systems with bioreactors to better mimic the dynamic *in vivo* environment and thus improve the accuracy of the *in vitro* models used.^28^ Paired with this *in vitro* investigation is the subsequent analysis of astrocyte response, particularly since the *in vitro* environment is significantly more simplified than its *in vivo* counterpart. We urge the field to develop not only characterization techniques that can be used *in vitro* and *in vivo* but also ones that reflect an understanding of the complexity of the astrocyte inflammatory response. Although characterizing inflammation in the body by the presence and number of astrocytes can give an indication of the existence and possibly the severity of inflammation, it is unhelpfully simplistic in understanding the implications and potential therapeutic targets of such inflammation. To fully understand inflammation, cells involved should be characterized by their temporal and spatial phenotypes, not just their numbers or presence, as previously expressed in Ref. 76. Adding to the complexity of understanding astrocytes and their role in inflammation, the possibility of different sub-types of reactive astrocytes needs to be recognised. Molecular and genomic analyses can provide insight into the different phenotypes and subsequent sub-types of astrocytes after injury. For example, genomic analysis of reactive astrocytes in either a stroke (middle cerebral artery occlusion, MCAO) or systemic endotoxin injection (LPS) model of neuroinflammation revealed that the gene expression profile is dependent on the injury stimuli.^77^ Although a core set of genes were expressed amongst reactive astrocytes in both models (GFAP, vimentin, Lcn2, Serpina3n), nestin was inducted sevenfold in the MCAO, whilst no induction was observed in the LPS model. The expression of nestin was restricted to astrocytes near the lesion core, whilst reactive astrocytes expressing GFAP were found in more distal regions, as well as near the lesion core. Differences in gene expression and the localisation of well-established markers for reactive astrocytes clearly demonstrate the heterogeneity of reactive astrocytes.^77^ Thus, there is an imperative for the scientific community to thoroughly characterize the phenotype of astrocytes after injury, using genomic and molecular analytical tools, as well as the incorporation of morphological and scar dimension analyses. The comprehensive characterization of reactive astrocyte phenotypes and sub-types can elucidate potential therapeutic targets to control the astrocyte response after injury and promote functional regeneration.

Developing biomaterial systems that can address inflammation after TBI provides the necessary foundation on which regenerative strategies can be developed to revolutionise functional recovery outcomes after TBI. Although there are many avenues of investigation required before an effective TBI treatment can be employed in the clinic, using tissue engineering is an exciting approach and will require the productive and innovative collaboration between biologists, tissue engineers, and clinicians to prove successful.
